# The role of independent walking in the maturation of the spinal locomotor output in children with cerebral palsy

**DOI:** 10.3389/fphys.2026.1828195

**Published:** 2026-05-13

**Authors:** Priscilla Avaltroni, Yury Ivanenko, Margherita Villani, Giulia Scordo, Francesca Sylos-Labini, Alessandra Medici, Carla Assenza, Daniela Morelli, Francesco Lacquaniti, Germana Cappellini

**Affiliations:** 1 Laboratory of Neuromotor Physiology, Istituto di Ricovero e Cura a Carattere Scientifico Fondazione Santa Lucia, Rome, Italy; 2 Department of Systems Medicine and Center of Space Biomedicine, University of Rome Tor Vergata, Rome, Italy; 3 Department of Pediatric Neurorehabilitation, Istituto di Ricovero e Cura a Carattere Scientifico Fondazione Santa Lucia, Rome, Italy

**Keywords:** cerebral palsy, gait rehabilitation, independent walking, neuromuscular modules, spinal locomotor output

## Abstract

The first two years of life are critical for the normal development of locomotor circuits. The emergence of independent walking (IW) during this period may reveal how the functional state of neural circuits develops in children with cerebral palsy (CP). In this cross-sectional study, we analysed the activation of eighteen lower limb muscles, corresponding neuromuscular modules, and spinal maps of motoneuron activity in 34 children with CP, in comparison with typically developing (TD) children. Consistency of common modules was used as a criterion for assessing the development of modularity of muscle activity patterns. We found a lack of maturation in children with CP prior to IW, which began late or never. These children exhibited diffused loci of motoneuron activity across lumbosacral segments prior to IW. They also showed two main basic modules, much like newborns, which were augmented to four main modules after IW. The findings indicate that, for IW, the developing spinal circuitry necessitates a differentiation of proximal and distal extensor activity, and an increase in the number of neuromuscular modules under the influence of corticospinal inputs. Early impairments in CP involve conservation of low dimensional control of locomotor movements until IW, suggesting a complex interplay between the maturation of neuromuscular modules and locomotor experience during a critical period of development.

## Introduction

For a long time, researchers have been deeply interested in the question of what is innate, and what is learned from experience during an infant’s early development (Cheron et al., 2001b, 2001a; Grillner and Wallén, 2004; Ivanenko et al., 2007; Dominici et al., 2011; Lacquaniti et al., 2013; Yang et al., 2019; Hinnekens et al., 2023; Cheung et al., 2024). The mechanisms and therapies behind early injury to brain motor pathways, including the corticospinal tract, make this issue especially relevant in the context of developmental conditions like CP whose gait abnormalities range from mild impairment to the need for walking aids (Graham et al., 2016). For these individuals, gait remains an essential concern.

The first two years of life are an important period of central nervous system maturation, during which spinal and supraspinal locomotor circuits, as well as sensorimotor integration gradually advance. Animal research provides evidence that postnatal damage of descending inputs disrupts spinal circuitry development and excitatory-inhibitory balance, especially during the critical period of motor development (Clowry, 2007; Friel et al., 2014). The positive impact of spinal cord stimulation further supports the role of the spinal cord in CP (Hastings et al., 2022). Thus, while brain injury is the primary cause of CP (Dan et al., 2025), much of the locomotor dysfunction may be related to the impaired state of the spinal cord (Levchenkova and Semenova, 2012; Yang et al., 2013; Noble, 2014; Cappellini et al., 2016; Trevarrow et al., 2021). Therefore, a better understanding of the spinal circuits involved in locomotor pattern generation in infancy may contribute to addressing developmental motor problems such as CP.

One way to study the development of locomotor pattern generators is to look backward from the periphery to what is being encoded in the central nervous system. This can be done by examining changes in the structure of muscle activity patterns and the spinal locomotor output. In this regards, it is now well established that locomotion involves modular spinal drives generating a set of basic temporal patterns of motoneuron activation with corresponding muscle synergies (Lacquaniti et al., 2012; Ting et al., 2015).

Several studies showed that, compared to TD children, children with CP use simpler motor control strategies with extensive co-activations of lower limb muscles (Steele et al., 2015; Goudriaan et al., 2022). However, little is known about the modular spinal drives around IW. Recently, Bekius et al. (2021) examined the structure of muscle synergies prior to and following IW. They found that infants with CP had impaired and fewer synergies than TD children. However, Bekius et al. (2021) did not primarily focus on the first independent steps or longitudinal recordings.

The present study examined spinal locomotor drive maturation early in development throughout the emergence of IW in children with CP (0.5–4 years old), by considering the common neuromuscular modules (Sylos-Labini et al., 2020) and investigating their neuromechanical correlates. For this purpose, we took special care to record independent steps within one week after IW onset. For comparison, neuromuscular modules were evaluated in TD children at IW onset, and in older children with severe CP who did not develop IW. Finally, since the motoneurons in the spinal cord are topographically organized in columns, we used an indirect spinal motor imaging technique (Ivanenko et al., 2013) to evaluate changes in lumbosacral pattern generation output.

We hypothesized that, prior to IW and independently of when this occurred, children with CP would conserve the modular structure with low dimensionality observed in TD children at birth and in the first few months afterwards (Dominici et al., 2011; Sylos-Labini et al., 2020; Hinnekens et al., 2023). Given that children with CP may develop IW much later than TD children, the low dimensional modular control would be maintained over an extended period of time after birth in these children.

However, new neuromuscular modules (including new temporal patterns and muscle synergies) would be added also in children with CP right after IW, making the overall control dimensionality comparable to that of TD toddlers. This hypothesis stems from the observation that, in TD toddlers, neural oscillations synchronous between the motor cortex and spinal motor neuron pools appear right after IW, in coincidence with the emergence of the new neuromuscular modules, consistent with the idea that corticospinal activity underlies these new modules (Smith et al., 2017; Zandvoort et al., 2022; Koster et al., 2025). Since work in animal models showed that the development of the corticospinal system depends on motor experience (Martin et al., 2004; Chakrabarty et al., 2009), we further predicted that older children with severe CP who never developed IW would not show the new neuromuscular modules presumably linked with the corticospinal influence on spinal motor drives, since these children lacked the specific motor experience of independent walking.

Therefore, our hypothesis assumes a reciprocal interaction between the development of the new neuromuscular modules and the locomotor experience gained with IW in children with CP, because they would both depend on the maturation of corticospinal influences and the integration of postural and locomotor control.

## Methods

### Participants

Twenty-eight children with a clinical diagnosis of CP (group 1, age-range 6–41 months, **Table 1**) were recruited from the Department of Paediatric Neurorehabilitation of Santa Lucia Foundation to investigate the emergence of IW. Nine typically developing children at the onset of IW were recruited by word of mouth (**Table 2**). In addition, we recruited six children with a clinical diagnosis of severe CP (group 2, 2.8–12 years, **Table 3**) who were unable to walk unsupported and presumably would never develop IW. All children with CP were enrolled in a rehabilitation program at the Paediatric Neurorehabilitation Department.

**Table 1:** Characteristics of children with cerebral palsy (group 1).

Subjects	Gender	GA, wk	BW, gr	Type of CP, SCPE	MAS at admission (R/L)	GMFM		GMFCS at ~2 yrs	Lesion type		
						At admission	at ~2 yrs		PWM	CDGM	M
CP1	F	27	750	dystonic bilateral	2/2	8.27	22.44	IV	x		
CP2	M	40	3380	dystonic bilateral	2/2	21.22	21.22	V			x
CP3	F	31	1400	spastic bilateral	0/0	22.45	68	II	x		
CP4	F	37	3240	spastic bilateral	2/1	22.05	47.44	IV	x		
CP5	F	38	3260	spastic monolateral	2/1	81.08	94.01	I			x
CP6	M	39	3100	ataxic	2/1	37.45	58.92	III			x
CP7	M	30	1560	spastic bilateral	2/1	35.03	99.16	I	x		
CP8	M	32	1700	ataxic	0/0	48.57	91.52	I	x		
CP9	F	30	1230	ataxic	0/0	47.57	90.61	I	x		
CP10	F	40	3870	spastic monolateral	0/1	21.33	79.59	I	x		
CP11	F	38	2695	spastic monolateral	2/0	23.71	94.37	I			x
CP12	F	39	2750	ataxic	0/0	44.97	81.5	I			x
CP13	M	43	3920	dystonic bilateral	2/2	10.40	32.6	IV			x
CP14	F	27	820	spastic bilateral	1/1	38.55	88.04	I	x		
CP15	M	25	700	spastic bilateral	0/0	19.55	20.12	IV	x		
CP16	M	39	2980	spastic monolateral	0/2	26.24	72.88	I		x	
CP17	F	30	1320	ataxic	0/0	14.00	45.9	III	x		
CP18	M	35	1860	spastic monolateral	1/0	38.18	87.6	I			x
CP19	M	36	2500	ataxic	0/0	55.34	85.4	I	x		
CP20	F	26	900	spastic bilateral	0/0	38.18	87	II	x		
CP21	F	30	1410	spastic bilateral	1/1	33.12	88.05	I	x		
CP22	M	25	940	ataxic	0/0	57.47	88.04	I	x		
CP23	F	33	2470	spastic monolateral	1/0	70.13	91	I			x
CP24	F	35	2340	spastic bilateral	2/1	39.59	86.68	III	x		
CP25	M	31	1400	spastic monolateral	1/1	58.21	90.01	I	x		
CP26	M	38	3300	ataxic	0/0	68.88	94.04	I	x		
CP27	M	30	2600	spastic bilateral	1/1	50.05	86.02	I			x
CP28	M	26	1145	spastic monolateral	1/1	22.44	41.20	I			x

PWM, Periventricular White Matter lesions; CDGM, Cortical and Deep Grey Matter lesions; M, Miscellaneous (white and grey matter lesions); GA, gestational age at birth; BW, birth weight; MAS Modified Ashworth Scale (the rater graded each ankle plantarflexion spasticity); SCPE, Surveillance of Cerebral Palsy in Europe; GMFM, Gross Motor Function Measure System was evaluated both at admission and at about 2 yrs of corrected age; GMFCS, Gross Motor Function Classification System was evaluated at about 2 yrs of corrected age.

To assess motor function, we used the Surveillance of Cerebral Palsy in Europe (SCPE) (Himmelmann et al., 2017), the Gross Motor Function Measure (GMFM), and the Gross Motor Function Classification System (GMFCS) (Palisano et al., 2006).

**Table 2:** Characteristics of typically development children recorded at the onset of their independent walking.

Subjects	Gender	GA, wk	BW, gr	Age, mo
TD1	M	39	3000	12
TD 2	F	39	3100	12
TD 3	M	39	3050	12
TD 4	F	40	2670	12
TD 5	F	38	3500	12
TD 6	F	39	3400	13
TD 7	F	40	3000	14
TD 8	M	38	3040	14
TD 9	M	38	3430	14

GA, gestational age at birth; BW, birth weight.

**Table 3:** Characteristics of children with severe cerebral palsy who did not develop IW (walked only with walker) (group 2).

Subjects	Gender	GA, wk	BW, gr	Type of CP, SCPE	MAS (R/L)	GMFM	GMFCS	Lesion type			Recorded session		
											Age, yrs	Treadmill	Overground
								PWM	CDGM	M		speed (#strides)	speed (#strides)
CPw1	M	35	1111	spastic bilateral	3/3	34.89	IV	x			2.8	0.1 (219)	–
CPw2	F	39	3288	spastic bilateral	3/3	66.88	III		x		3.2	–	0.2 (82)
CPw3	M	43	3920	dystonic bilateral	2/2	32.60	V			x	3.8	0.1 (210)	–
CPw4	F	31	1580	spastic bilateral	2/2	48.44	IV	x			6.5	0.1 (32)	–
CPw5	F	42	2700	spastic bilateral	3/2	40.50	IV			x	11	–	0.13 (32)
CPw6	F	31	1580	spastic bilateral	2/2	42.10	IV	x			12	0.05 (54)	–

PWM, Periventricular White Matter lesions; CDGM, Cortical and Deep Grey Matter lesions; M, Miscellaneous (white and grey matter lesions); GA, gestational age at birth; BW, birth weight; MAS Modified Ashworth Scale (the rater graded each ankle plantarflexion spasticity); SCPE, Surveillance of Cerebral Palsy in Europe; GMFM, Gross Motor Function Measure; GMFCS, Gross Motor Function Classification System.

Since CP is defined primarily on a functional rather than etiological basis, inclusion criteria were based on the presence of a non-progressive disorder of movement and posture attributed to early disturbances in the developing brain (Dan et al., 2025). Diagnosis was established by experienced paediatric neurologists based on medical history, neurological examination, developmental trajectory, and brain magnetic resonance imaging (MRI) findings when available. Diagnostic procedures were consistent with current early detection recommendations (Novak et al., 2025), supporting reliable diagnosis within the first months of corrected age when converging clinical and neuroimaging findings indicate persistent motor impairment. Motor type classification was based on the predominant pattern of motor impairment, following internationally recognized criteria using the Surveillance of Cerebral Palsy in Europe, SCPE (Himmelmann et al., 2017), distinguishing unilateral and bilateral spastic forms, as well as dyskinetic and ataxic presentations where applicable. Brain MRI had been performed as part of routine clinical evaluation. Structural abnormalities were classified according to established neuroimaging criteria (Himmelmann et al., 2017), including periventricular white matter injury, cortical and deep grey matter lesions, and other maldevelopments or miscellaneous lesions (see **Table 1**). Gross motor function was evaluated using the Gross Motor Function Classification System (GMFCS; Palisano et al., 2006). When available, Gross Motor Function Measure (GMFM-88) assessments were performed by experienced physiotherapists according to standardized procedures. Ankle plantarflexor muscle spasticity was evaluated by the Modified Ashworth Scale (MAS) (Bohannon and Smith, 1987) (**Table 1**). Exclusion criteria included progressive neurological disorders, known genetic syndromes affecting motor development, lower extremity orthopedic surgery within the previous year, and botulinum toxin injections within the preceding four months.

To avoid acute effects of therapy, no rehabilitation was administered during the two days preceding the recordings. Moreover, when children were first admitted to the unit, rehabilitation had not yet been initiated. The Ethics Committee of Santa Lucia Foundation approved the study procedures (protocol n.CE/PROG.875) that adhered to the Declaration of Helsinki for medical research, and informed written consent was obtained from the parents.

### Protocol and data recording

**Figure 1:**
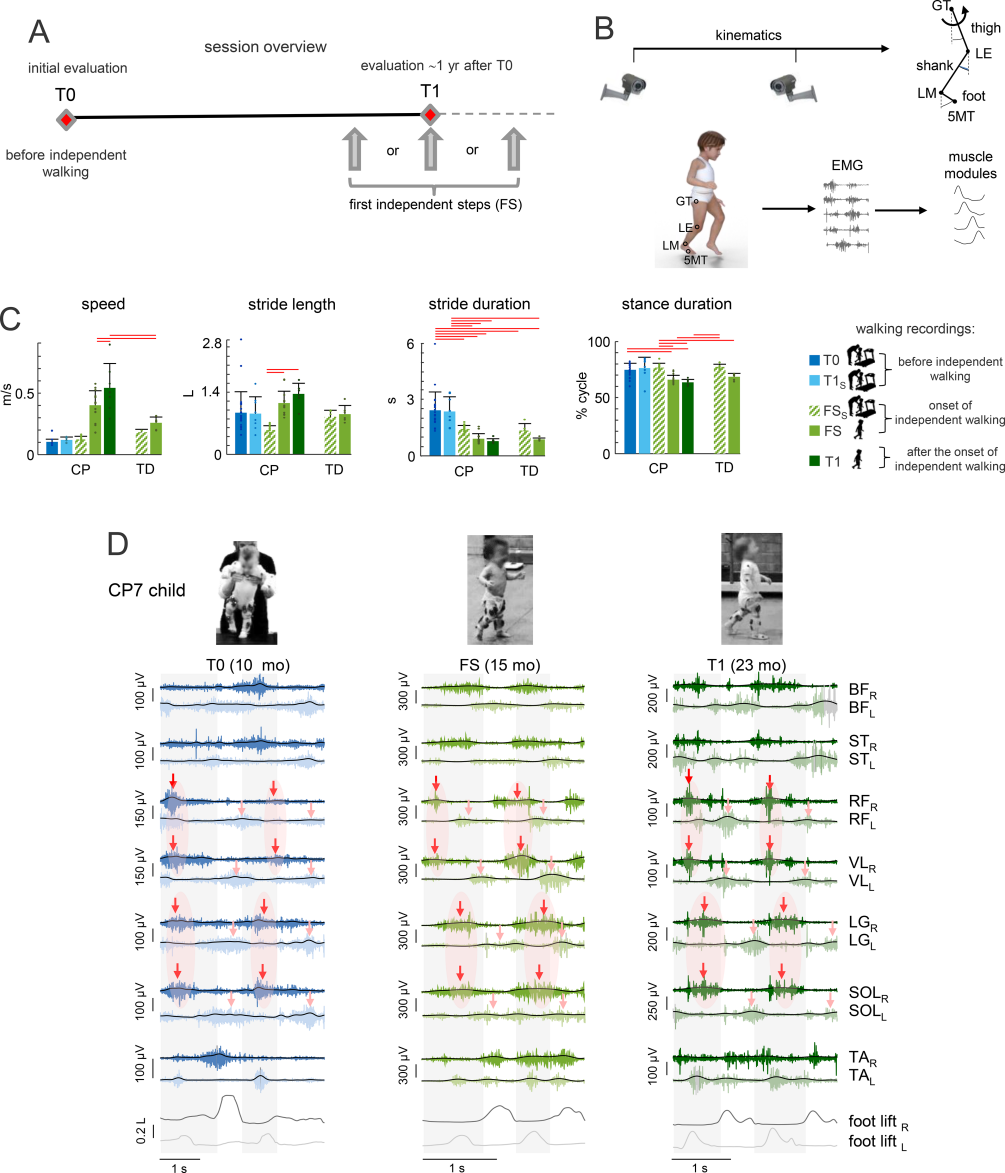
Protocol and gait characteristics. **(A)** Schematic session overview. Gait analyses were performed during initial evaluation before the onset of independent walking (T0), ˜1 yr after (T1), and during first independent steps (FS) that could occur either before or after T1. **(B)** Schematic view of performed recordings. Recorded EMG signals were used to analyse muscle modules and spinal maps of MN activation. **(C)** General gait parameters (walking speed, stride length, cycle duration and stance duration) in different walking recordings of CP children and in TD children. Stride length was normalized to the limb length L (thigh + shank). Horizontal lines denote significant differences between groups (Tukey’s HSD, p<0.05). For walking speed (left panel), we reported significant differences only for unsupported walking. **(D)** Examples of EMG activity in a child (CP7) before IW (left), at FS (middle) and after the onset of IW (right). Muscle activity is shown during two consecutive strides, grey areas denote the stance phase of the right leg. Foot lift (fifth metatarso-phalangeal joint marker) is also shown on the bottom. L, left, R, right, RF, rectus femoris, VL, vastus lateralis, BF, biceps femoris (long head), ST, semitendinosus, LG, gastrocnemius lateralis, SOL, soleus, TA, tibialis anterior. Red arrows and areas approximately indicate the major activity of extensor muscles (RF, VL, LG, SOL) of the right leg. Radish arrows indicate it for the left extensors.

Walking in children with CP (group 1) was tested longitudinally at different time points (**Figure 1A**):

- at T0: when they were first admitted to the Paediatric Neurorehabilitation Department, before IW onset;
- within a week after IW onset when they took their first unsupported steps (FS);
- at T1: ~one year after T0.

At T0, they could not walk independently (except for CP21) and were supported during stepping on a treadmill. In order to accomplish this, the child was held beneath the armpits by an experienced physiotherapist, with the soles of the feet touching the surface of a paediatric treadmill (model 3 Carlin’s Creations, Sturgis, MI, 81x46 cm length x width), and the parent or experimenter encouraged the child to take steps. The treadmill speed (range 0.05-0.2 m/s) was chosen to be comfortable for infants. At FS (that could occur either before or after T1, **Figure 1A**), we recorded unsupported overground stepping (~8 m) at a self-selected speed. Moreover, for comparison with stepping at T0, we also recorded supported stepping on a treadmill (FS_S_). At T1, children able to walk independently were recorded during unsupported overground walking. Otherwise, they were recorded during supported treadmill stepping (T1_S_) at 0.1-0.2 m/s, as at T0. **Table 4** reports the recorded walking conditions. Some of the missing data related to T0 and T1 in children with CP (**Table 4**) were attributed to participant dropout (CP8, CP15 at T1), dropout after recording of supported stepping during the experiment (CP12 and CP16 at FS), or not achieving yet T1 (CP4 and CP21 at T1). Five children with CP (CP21, CP25-CP28) were recorded only during the first unsupported steps.

**Table 4:** Recorded sessions in children with cerebral palsy (group 1).

Subjects	T0 (before FS)		FS			T1 (∼1 yr after T0)		
	Age, mo	Supported, treadmill	Age, mo	Supported, treadmill	Unsupported, overground	Age, mo		Speed (#strides)
		Speed (#strides)		Speed (#strides)	Speed (#strides)			
CP1	6	0.1 (52)	–	–	–	20	before FS	0.1 (21)
CP2	7	0.1 (87)	–	–	–	19	before FS	0.1 (23)
CP3	8	0.1 (32)	–	–	–	20	before FS	0.1-0.15(116)
CP4	9	0.05-0.1 (23)	–	–	–	–	–	–
CP5	10	0.2 (17)	22	0.1 (203)	0.35 (64)	22	FS	
CP6	10	0.1 (15)	–	–	–	23	before FS	0.15 (9)
CP7	10	0.1 (36)	15	0.1 (42)	0.37 (214)	23	after FS	0.51 (81)
CP8	10	0.1 (7)	–	–	–	–	–	–
CP9	11	0.1 (89)	15	0.1 (32)	0.48 (136)	23	after FS	0.47 (19)
CP10	11	0.1 (5)	–	–	–	22	before FS	0.1-0.2 (127)
CP11	12	0.1 (25)	–	–	–	28	after FS	0.67 (183)
CP12	14	0.1 (8)	27	0.1 (289)	–	27	FS	
CP13	14	0.1 (8)	–	–	–	26	before FS	0.1 (176)
CP14	14	0.1 (138)	20	0.1 (114)	0.58 (136)	27	after FS	0.9 (35)
CP15	15	0.1 (4)	–	–	–	–	–	–
CP16	16	0.1 (54)	21	0.1-0.15 (118)	–	27	after FS	0.38 (30)
CP17	17	0.1 (4)	29	0.1-0.2 (325)	0.42 (92)	29	FS	
CP18	18	0.1 (30)	30	0.15 (105)	0.25 (44)	30	FS	
CP19	18	0.1 (200)	23	0.1-0.2 (293)	0.55 (202)	30	after FS	0.58 (95)
CP20	22	0.1 (45)	41	0.1-0.2 (331)	0.47 (190)	35	before FS	0.1 (151)
CP21	–	–	22	0.1-0.15 (97)	0.53 (179)	–	–	–
CP22	23	0.1 (70)	29	0.1-0.2 (292)	0.43 (105)	35	after FS	0.30 (117)
CP23	24	0.1-0.15 (62)	37	0.1-0.2 (149)	0.25 (86)	37	FS	
CP24	25	0.1 (73)	–	–	–	37	before FS	0.1-0.15 (118)
CP25	–	–	15	–	0.18 (66)	–	–	–
CP26	–	–	17	–	0.43 (16)	–	–	–
CP27	–	–	20	–	0.33 (78)	–	–	–
CP28	–	–	20	–	0.35 (103)	–	–	–

T0, initial evaluation before the onset of independent walking; FS, first steps (onset of independent walking); T1, evaluation ∼1 year after T0.

The age refers to the children’s corrected age, the speed for overground walking (m/s) is the average speed over all strides. T0 or T1 could occur at around the time of FS, in such cases we indicated the characteristics of walking recordings in the FS column (and “FS” in the T0 or T1 column). If a child at T1 already started to walk independently, he/she was recorded during unsupported overground walking at a self-selected speed, otherwise, we recorded supported treadmill stepping at 0.1-0.2 m/s, as at T0.

For comparison, we also recorded walking in nine TD children (**Table 2**) at IW onset. TD children were recorded during unsupported (at ~0.2-0.3 m/s) and supported (at 0.05-0.2 m/s on a treadmill) walking conditions at similar walking speeds adopted for children with CP.

In additional experiments, we evaluated walking in children with severe CP (group 2, **Table 3**) who were unable to walk unsupported. Either overground walking with a walker and supported walking on a treadmill (with a walker or with physiotherapist assistance) were used for children with severe CP. This choice was made based on physiotherapist’s preferences or whether the child expressed his/her preferences. The number of strides included in the analysis (reported in **Tables 3**, **4**) was not predefined *a priori*, but was determined by the number of steps each participant was able to complete during the experimental sessions.

Walking kinematics was recorded at 200 Hz by means of Vicon-Nexus system (Oxford, UK) with 10 cameras placed around the walking path. Infrared reflective markers were attached on each side of the child to the skin overlying the following landmarks: hip joint (greater trochanter, GT), knee joint (lateral epicondyle, LE), ankle joint (lateral malleolus, LM) and fifth metatarso-phalangeal joint (5MT). Electromyographic (EMG) activity was recorded by means of surface electrodes simultaneously from the following 9 muscles from each body side: rectus femoris (RF), vastus lateralis (VL), vastus medialis (VM), biceps femoris (long head) (BF), semitendinosus (ST), gastrocnemius medialis (MG), gastrocnemius lateralis (LG), soleus (SOL), and tibialis anterior (TA). All EMGs were recorded at 2000 Hz using the wireless Trigno EMG system (Delsys Inc., Boston, MA), bandwidth of 20–450 Hz, overall gain of 1000. Sampling of kinematic and EMG data were synchronized.

### Data analysis

*Kinematics*. Gait cycle was defined as the time between two successive foot–floor contacts according to the local minima of the vertical displacement of the ankle (or 5MT in case of toe-walking) marker (Dominici et al., 2007). The timing of the lift-off was determined when the 5MT marker was elevated by more than 2 cm. For overground walking, walking speed for each stride was computed as the mean speed of the horizontal trunk (virtual marker located at the midpoint between left and right GT markers) movement. For walking on the treadmill, walking speed refers to the treadmill belt speed. For overground walking, the stride length was measured as the horizontal displacement of the 5MT marker, while on the treadmill, it was calculated as the belt speed multiplied by the stride duration. The stride length and foot trajectory were normalized to the limb length (L, thigh + shank). Data were time-interpolated over individual gait cycles to fit a normalized 200-point time base.

We also assessed the intersegmental coordination by analysing the temporal changes of the elevation angles at the foot, shank, and thigh, that have been shown to covary in healthy adults and older children during walking (Bianchi et al., 1998; Hallemans and Aerts, 2009; Degelean et al., 2012; Cappellini et al., 2016). Plotting these angles in three dimensions for each gait cycle describes a path that may be leastsquares fitted to a plane. To assess the gait loop and related plane, we calculated the time-varying elevation angle covariance matrix across each gait cycle (after subtracting their mean value). The orthogonal directions of maximal variance correspond to the three eigenvectors, *u_1_–u_3_*, which are ranked according to their corresponding eigenvalues. The bestfitting plane of angular covariation is where the first two eigenvectors, *u_1_* and *u_2_*, are located. The normal to the plane, or *u_3_*, is the third eigenvector and determines the direction of the plane. We examined the *u_3t_* parameter (the direction cosine of the normal to the plane with the axis of thigh elevation) in order to measure the rotation of the plane (Bianchi et al., 1998; Dominici et al., 2007).

*Muscle activity*. The raw EMG signals were high-pass filtered (60 Hz), full-wave rectified and low-pass filtered at 5 Hz with a zero-lag fourth-order Butterworth filter to obtain envelope time series (Sylos-Labini et al., 2022). The EMG envelopes were averaged across all cycles for each participant separately for the ipsilateral leg (used to define the gait cycle) and contralateral leg. To characterize differences in the timing of EMG bursts, we also computed the centre of muscle activity (*CoMA*) using circular statistics, as the angle of the vector (1^st^ trigonometric moment) in polar coordinates (polar direction denoted the phase of the gait cycle, with angle *θ* that varies from 0 to 360^°^) that points to the centre of mass of that circular distribution of the muscle EMG using the following equations (Cappellini et al., 2016),


$$A=\sum_{t=1}^{200}\left(\cos\theta_{t}\times EMG_{t}\right)$$

$$B=\sum_{t=1}^{200}\left(\sin\theta_{t}\times EMG_{t}\right)$$

$$CoMA=\tan^{-1}\left(B/A\right)$$

where *t* = 1:200 is a normalized time base. The *CoMA* provides an estimate of the timing of the EMG bursts.

*Basic muscle activation patterns*. We examined muscle synergies and common temporal activation patterns that compose basic neuromuscular modules (Lacquaniti et al., 2013; Cheung et al., 2020; Sylos-Labini et al., 2022). In line with the modular control framework, we distinguish between temporal and spatial components of muscle activation. Basic activation patterns (also denoted as activation coefficients in the literature on muscle synergies, e.g. Cheung et al., 2020) represent the temporal structure of the motor output, reflecting time-varying command signals emitted by the central nervous system. These signals are thought to recruit multiple α-motoneuron pools quasi-synchronously and to activate groups of muscles at specific phases of the motor task. The spatial component, instead, is represented by the weighting of each muscle within a given module (also denoted as time-invariant synergy vectors, e.g. Cheung et al., 2020), corresponding to the distribution of the activation pattern across muscles. Thus, each module can be described as a combination of a temporal activation pattern and its associated spatial muscle weightings, which together contribute to the generation of the observed EMG activity. In this framework, the EMG profile of each muscle results from the weighted combination of a limited number of basic activation patterns, consistent with the concept of modular organization of motor control.

First, the modules for each single stride were extracted from EMG profiles of 18 (bilateral) muscles using non-negative matrix factorization algorithm (Sylos-Labini et al., 2020). It was applied to model *EMGs* (*m* × *t* matrix) as a linear combination of basic activation patterns *P*(*t*) (*n* × *t* matrix) and weighting coefficients or muscle synergies *W* (*m* × *n* matrix):

(1) $$EMG=\sum_{i}^{n}P_{i}W_{i}+error,n\leq m$$

where *m* is the number of muscles and *n* is a predetermined number of basic patterns. To determine *n* for each stride, we kept the smallest number of *P*(*t*) that accounted for ≥80% of the variance of EMG. The choice of the VAF threshold (~80%) for individual strides is consistent with previous studies on locomotor modularity in both developing and clinical populations, where this level has been shown to capture the main structure of motor control while avoiding overfitting to noise (Sylos-Labini et al., 2020, 2022).

After identifying the main basic modules for each stride, we applied cluster analysis by pooling together all strides of all participants for each specific condition. The clustered patterns described in the subsequent analysis are used to identify groups of similar activation profiles across strides. These clusters can be interpreted as data-driven representations of recurring temporal activation patterns, each associated with a corresponding spatial distribution of muscle weights. In this sense, the clustering procedure allows the extraction of representative modules that reflect both the temporal organization and the spatial structure of muscle activity. This allowed us to identify common (consistent across strides and subjects) basic modules for a given walking condition.

To identify similar activation patterns, all *P*(*t*) (Equation 1) extracted from individual strides were pooled together and partitioned in *k* mutually exclusive patterns using the k-means algorithm. We determined the optimal number of clusters using the silhouette method. The silhouette value (*S*, range from -1 to +1) indicates how similar a particular data point is relative to the other data points in that cluster. When *S* was less than 0.2, all patterns of activation (or synergy weights) were considered mismatched and removed from the cluster (Sylos-Labini et al., 2020).

In addition to pooling the basic patterns of all strides and all subjects in the group and then performing k-means clustering, we performed a complementary analysis based on direct extracting the modules from the strides of individual subjects, as described in the **Supplementary Material**.

*Spinal maps of motoneuron activity*. The averaged patterns of EMG activity, providing an indirect measure of the net firing of motoneurons (MNs) of a muscle, were mapped onto the approximal rostrocaudal location of MNs-pools (Ivanenko et al., 2013). We used the non-normalized procedure (EMGs were expressed in μV). The MN activation in a particular segment *S_j_* was calculated as:

(2) $$S_{j}\left(t\right)=\;\frac{\sum_{i=1}^{n_{j}}\;k_{ij}\;.\;EMG_{i}\left(t\right)\;}{n_{j}}$$

where *n_j_* is the number of *EMG_i_* waveforms corresponding to the *j_th_* segment and *k_ij_* is the weighting coefficient for the *i_th_* muscle (Ivanenko et al., 2013). We also calculated the centre of activity (*CoA*) of the six (from L2 to S2) most active lumbosacral segments (Ivanenko et al., 2013), and using the averaged *CoA* of TD children at their FS as a reference, we correlated the *CoA* of individual subjects in the different groups of children with the averaged *CoA* of TD children.

### Statistics

Descriptive statistics included the calculation of the mean and standard deviation (SD). One-way ANOVA was used to evaluate the effect of group on different variables. Tukey HSD *post-hoc* test was used to detect significant differences between groups by taking into account multiple comparisons. Statistics on correlation coefficients was performed on *Z*-transformed values. To characterize differences in the timing of EMG bursts, we computed the centre of muscle activity (*CoMA*) (Cappellini et al., 2016) using circular statistics. The Watson-Williams test with Bonferroni correction was used for these *post-hoc* comparisons. Reported results are considered significant for *p* < 0.05.

## Results

### General walking and muscle activity characteristics

We first report the results of children with CP group 1. They showed greater variability in IW onset (15–41 mo) than TD children of our sample (12–14 months, **Supplementary Tables**). General gait characteristics are shown in **Figure 1C**. Prior to IW (at T0), children with CP stepped supported on a treadmill in a limited range of speeds (0.05-0.2 m/s). Right after IW onset (FS), they were able to walk unaided on the ground, and the average walking speed increased from FS to T1 (0.40 ± 0.12 vs. 0.54 ± 0.20 m/s, respectively, *p* < 0.01 Tukey HSD, η^2^ = 0.38).

**Figure 2:**
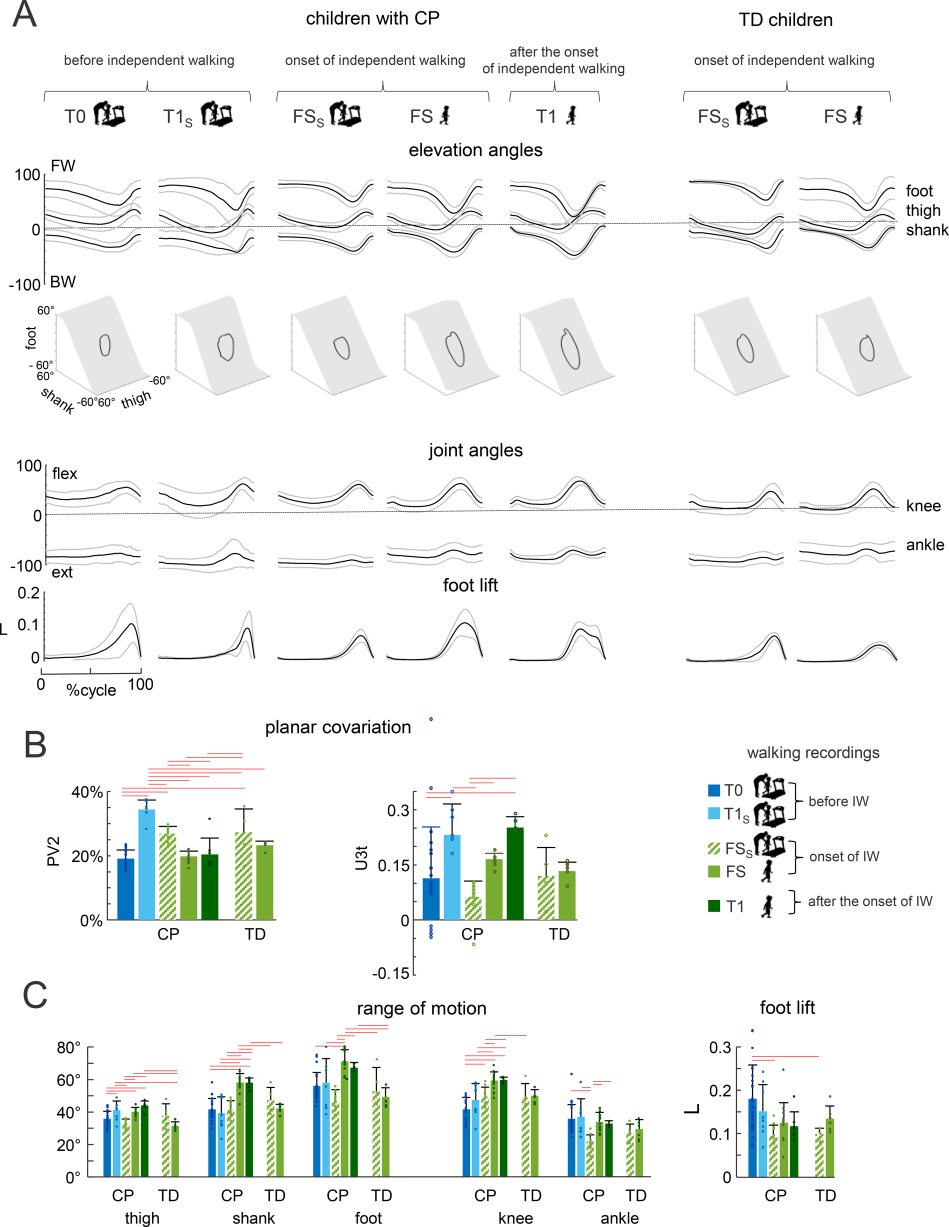
Walking kinematics in different groups of children. **(A)** Ensemble-averaged (mean ± SD) foot, thigh and shank elevation angles, knee and ankle joint angles, and vertical foot (5MT) movements plotted vs. normalized gait cycle. Ensemble-averaged 3-dimensional gait loops and interpolation planes are shown in the middle. Gait loops are obtained by plotting the thigh vs. shank and foot waveforms (after mean values subtraction). Gait cycle paths progress in time in the counter-clockwise direction, touch-down and toe-off phases corresponding roughly to the top and bottom of the loops, respectively. The interpolation planes result from orthogonal planar regression. Children with CP at T0, T1S and FS and TD children at their FS frequently moved the leg in such a way that the foot lift had only one maximum at mid-swing, while children with CP at T1 have two peaks and a minimum foot clearance during mid-swing, in accordance with the development of the pendulum mechanism of walking after the onset of IW. **(B)** Percentage of total variation explained by 2^nd^ principal component (PV2) and u3t parameter that characterizes the orientation of the normal to the plane (mean+SD). **(C)** Ranges of angular motion and vertical foot (5MT) excursion. Foot lift was normalized by the limb length (L) Horizontal lines denote significant differences (One-way ANOVA with Tukey’s HSD, p<0.05).

**Figure 2A** illustrates ensemble-averaged (± SD) segment elevation and joint angles. Elevation angles showed a relatively wide range of oscillations necessary for stepping, variability was partly caused by differences in the walking speed. In all children, temporal changes of the elevation angles covaried along a plane (*u_1_* parameter was on average<1.5% for all groups), describing a characteristic loop over each stride (**Figure 2A**). The width of the loop (characterized by u_2_) and the orientation of the covariance plane (characterized by u_3t_) were comparable across the groups (**Figure 2B**). Thus, the planar covariation of elevation angles, which is a kinematic ‘signature’ of stepping (Ivanenko et al., 2008), was present in all groups of children. Range of motion values showed variability across groups (**Figure 2C**), which may be partly influenced by differences in walking speed. Foot lift (**Figure 2C**) showed notable inter-individual variability, with higher values observed before IW, likely reflecting the less refined motor control.

**Figure 3:**
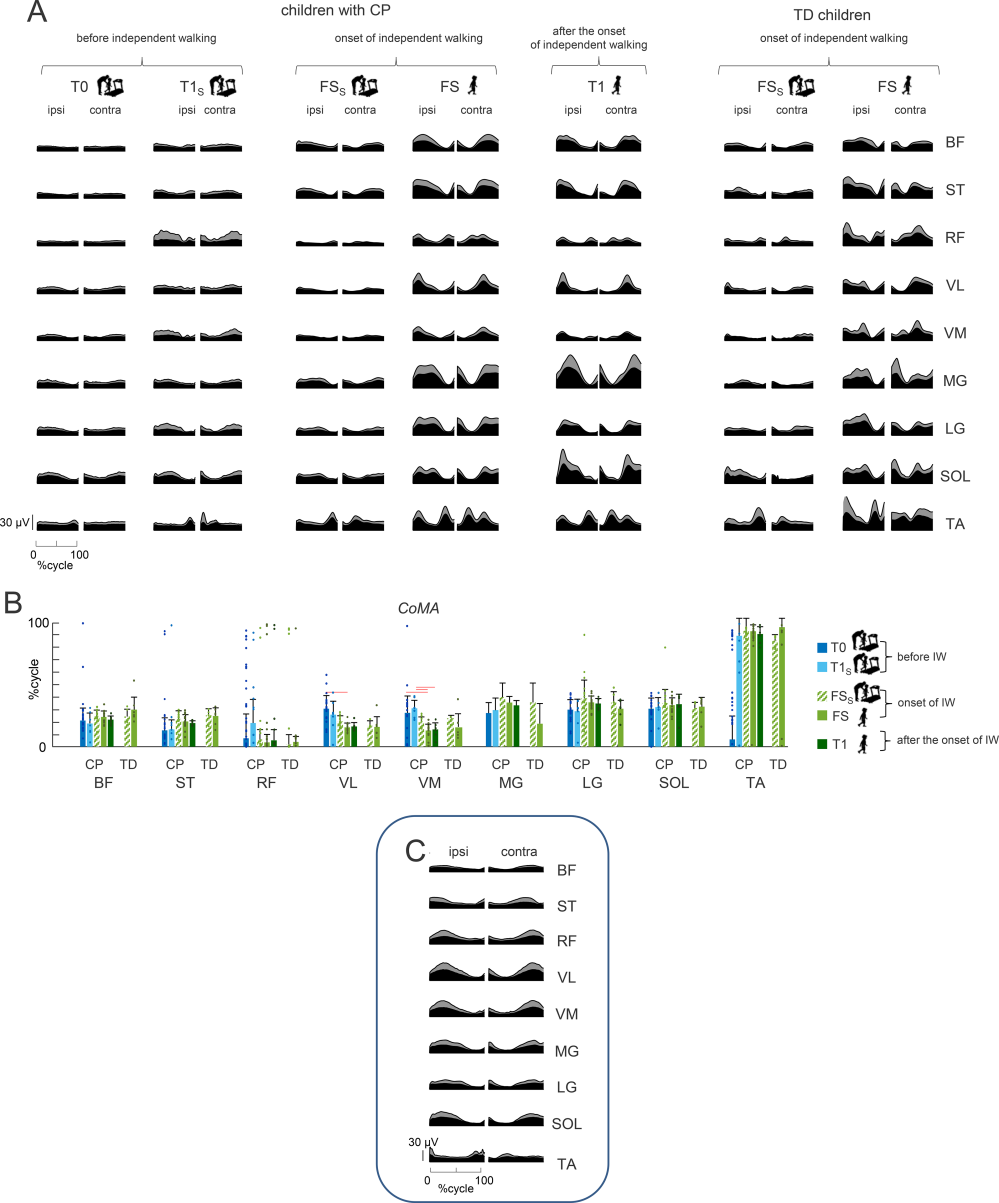
Characteristics of muscle activity. **(A)** Ensemble-averaged (mean+SD) EMG envelops of 18 bilateral leg muscles recorded in children with CP (group 1) and TD children. EMG data are plotted vs. normalized gait cycle. **(B)** CoMA of EMG envelops (means+SD). Horizontal lines denote significant differences between groups (Watson–Williams multisample test with Bonferroni correction, p<0.05). **(C)** Ensemble-averaged EMG envelops for children with severe CP that can walk only with external aids (group 2). RF, rectus femoris, VL, vastus lateralis, VM, vastus medialis, BF, biceps femoris (long head), ST, semitendinosus, MG, gastrocnemius medialis, LG, gastrocnemius lateralis, SOL, soleus, TA, tibialis anterior.

**Figure 1D** illustrates an example of EMG activity patterns in a child with CP before (T0), just after IW onset (FS), and several months after IW (T1). At T0, distal and proximal extensor muscles were quasi-synchronously activated. By contrast, at FS and T1, the distal extensors were activated around late stance and the proximal extensors at the beginning of stance. **Figure 3** illustrates ensemble-averaged EMG patterns of all recorded muscles of all groups showing similar trends. The higher intensity of EMGs at FS and T1 (expressed in μV, **Figure 3A**) was likely associated with the faster walking speed, however, one can also see essential differences in the extensor waveforms. All of the recording sessions generally showed a noticeable level of inter-stride variability (that could be observed by simple inspection of EMG signals, e.g., **Figure 1D**, though it was not explicitly quantified), which is normal for an infant’s stepping (Teulier et al., 2012; Sylos-Labini et al., 2022).

### Development of basic muscle activity modules during the emergence of IW

**Figure 4:**
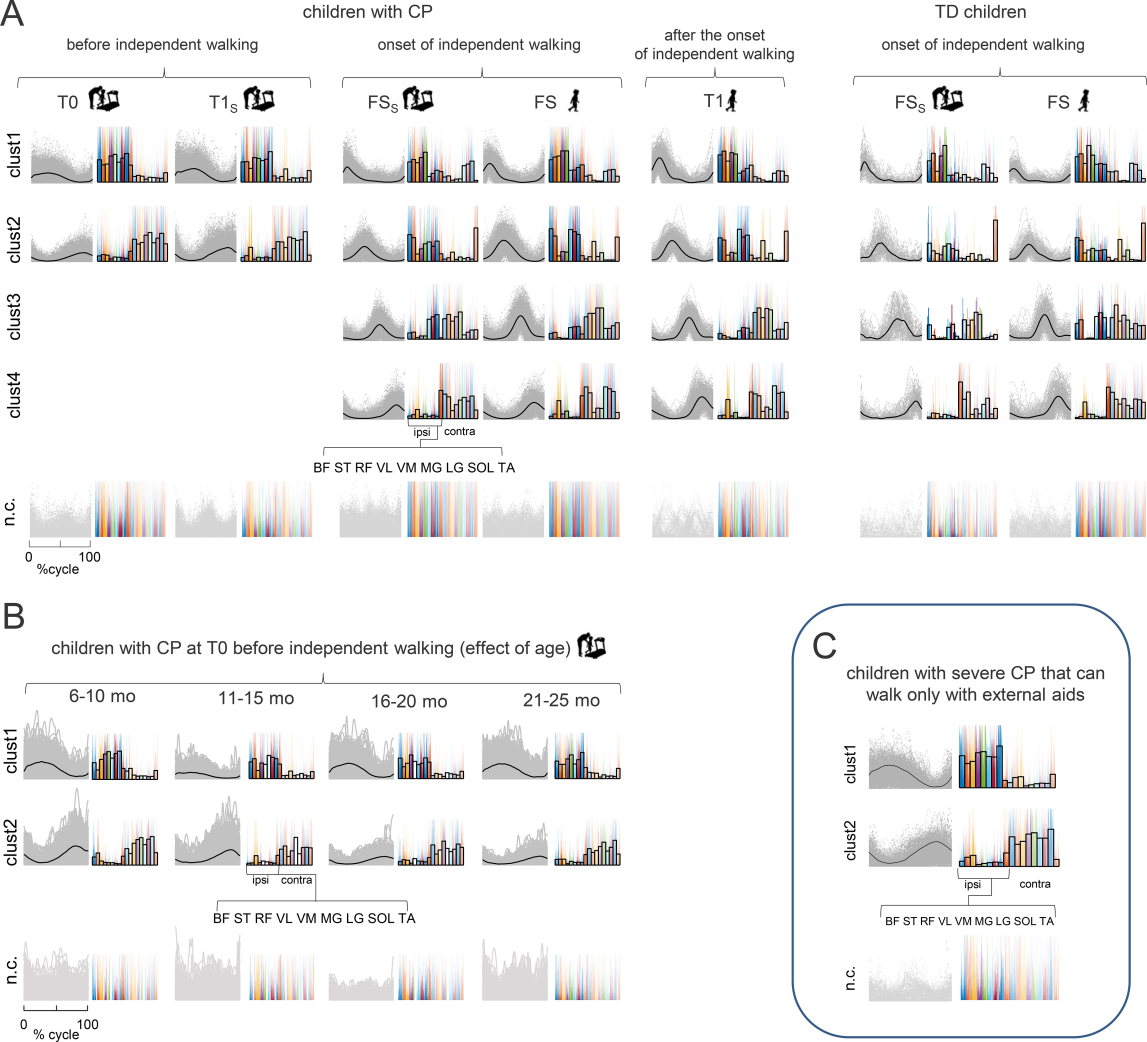
Neuromuscular modules of bilateral EMGs. **(A)** Clusters of activation patterns (silhouette values S > 0.2) from single strides of all subjects (CP group 1, and TD children) are shown in grey, average patterns in black. Prior to applying non-negative matrix factorization, we normalised the EMG amplitude of each muscle to the maximum over all strides of a given participant after subtracting the minimum over the stride. For each subject group, the resulting clusters of basic activation patterns P were ordered chronologically, based on the timing of the main peak in relation to the step cycle. Corresponding synergies weights are shown on the right for single strides in colour, average values as empty bars. bottom: Not-clustered (nc, S ≤ 0.2) activation patterns (light grey) and associated weights. **(B)** Neuromuscular modules of children with CP before independent walking (T0 evaluation). Children were divided into four groups (6–10 mo, 11–15 mo, 16–20 mo, and 21–25 mo). This was done to test if age before IW had any bearing on the complexity (dimensionality) of muscle activation patterns. The same format as in **Figure 5**. **(C)** Clusters of basic activation patterns in children with severe CP that can walk only with external aids (walkers) (group 2).

We evaluated the complexity of muscle coordination and modular engagement of groups of muscles (Lacquaniti et al., 2013). At T0, we found two optimal clusters of basic activation patterns. The same two clusters were still observed in children who did not accomplish IW when tested at T1_S_, about a year after T0 (**Figure 4A**, left panel). However, four optimal clusters were identified in children tested at FS and FS_S_, that is, after they accomplished IW. Roughly similar four clusters were still present at T1, as well as in TD children right after IW (**Figure 4A**). **Supplementary Figure S1** shows silhouette values of corresponding weights (synergies). For this analysis, we pooledtogether the strides of all participants in a specific group. By calculating the proportion of eachparticipant’s strides for every activation pattern and synergy, we found a comparatively consistent distribution of clusters and synergies among participants for every session, indicating that each activation pattern and synergy was present in every child (**Supplementary Figure S2**).

In addition, we performed a complementary analysis based on directly extracting the modules fromthe strides of individuals (in children with CP who performed sufficiently large number of stridesfor such analysis) and we found an increase in muscle synergy dimensionality after the onset of IW (**Supplementary Figure S3A**). By further exploring the individual modules by extracting them from averaged EMG data (see **Supplementary Material**), we also did not find significant correlation (r=-0.04, p=0.87) between the clinical score at admission (GMFM, **Table 1**) and the percent of variance accounted by 2 modules (the number prevailing at T0),suggesting that at T0 most children exhibited similar reduced dimensionality. In group 2, theindividual subject analysis also showed reduced dimensionality (2 modules, **Supplementary Figure S3B**).

IW occurred comparatively late in children with CP (see above). Accordingly, their age at T0 varied considerably, between 6 and 24 months (**Table 4**). To test if age affected the complexity of muscle activation patterns prior to IW, we applied cluster analysis separately for different age groups, and found that the two activation patterns were present in all age groups until IW onset (**Figure 4B**).

### Spinal maps of motoneuron activity

**Figure 5:**
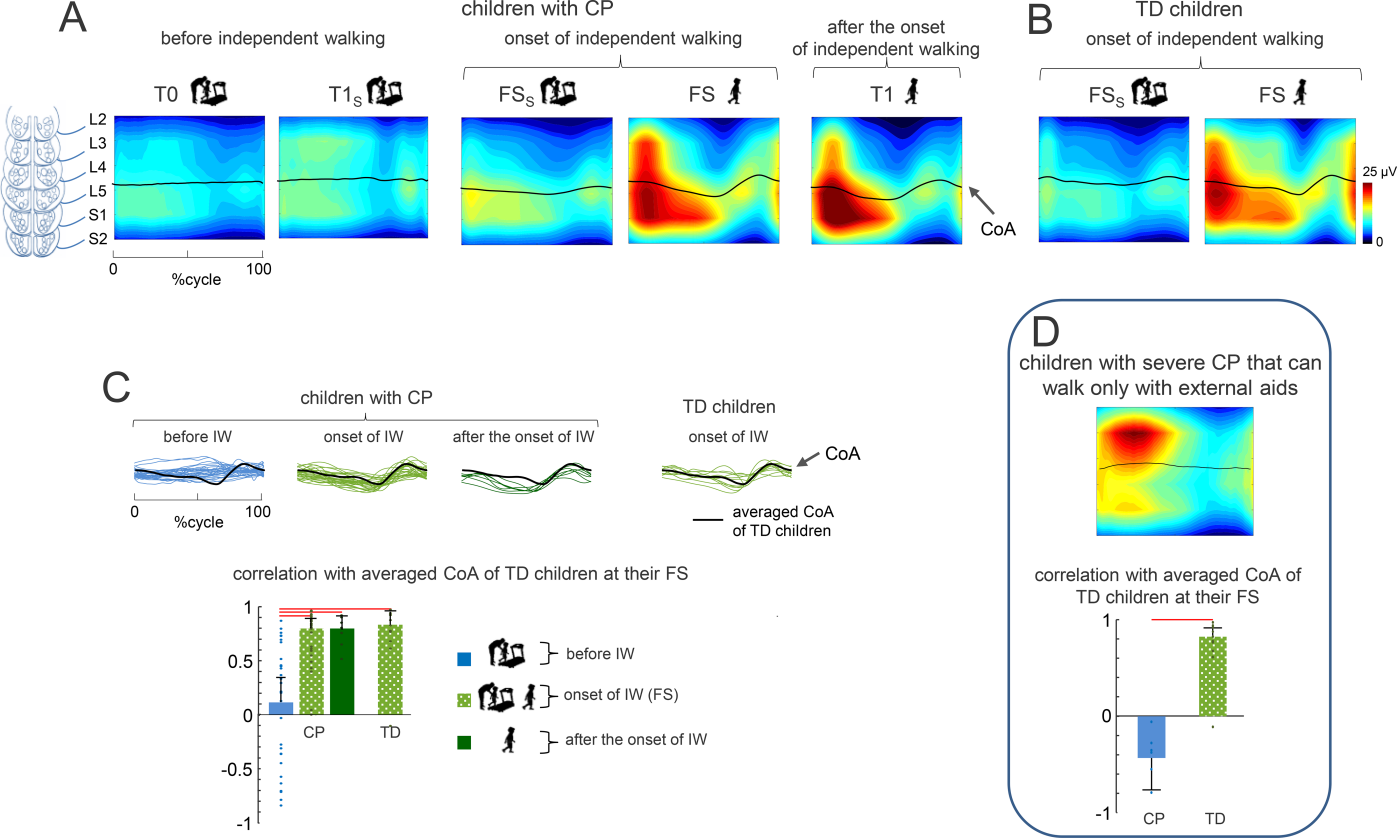
Spinal maps of MN activity during stepping. **(A)** Spatiotemporal unilateral maps of MNactivity in children with CP (group 1) as a function of gait cycle and spinal segment level(L2−S2) were reconstructed using the averaged EMG envelopes of the ipsilateral leg (**Supplementary Figure S2**) and location of MNs-pools in the human spinal cord. Even though we recorded a limited set of muscles, we have previously shown that the muscles recorded here are those that contribute mostly to the overall spinal maps (Ivanenko et al., 2013). To visualize a continuous smoothed rostrocaudal spatiotemporal activation of the spinal cord, we used a filled contour plot that computes isolines calculated from segment activation waveforms and fills the areas between the isolines using separate colours. The black curves refer to the centre of MN activity (*CoA*). **(B)** The same for TD children. **(C)** Correlation (mean+SD) between the averaged *CoA* of TD children at their FS and the *CoA* of individual subjects in the different groups of children. Data from sessions prior to IW were pooled together (T0+T1_S_), as well as data from FS (FS+FS_S_). Horizontal lines denote significant differences (One-way ANOVA with Tukey’s HSD, *p* < 0.05). On the top panels, CoA traces of individual subjects are shown along with averaged CoA of TD children (in black). Note very poor correlations of lumbosacral oscillation of MN activity (*CoA*) prior to IW in children with CP. **(D)** Spinal locomotor output in children with severe CP that can walk only with external aids (group 2).

Using the spinal motor imaging technique, we assessed the topography of spinal locomotor pattern generation using Equation 2 for evaluating MN activity in each segment (**Figure 5A**). In children with CP, the main loci of MN activity were prolonged during the gait cycle and widely spread across lumbar and sacral segments prior to IW. After IW, they became more localized, exhibiting a distinct oscillation of the *CoA* between the lumbar (at stance onset) and sacral (late stance) segments. This trend could also be seen in the spinal maps and *CoA* of individual subjects (**Supplementary Figure S4A**).

We estimated the similarity of *CoA* oscillations of individual children with CP with averaged *CoA* of TD children at their FS (**Figures 5B, C**), and found poor correlations (0.11 ± 0.24) prior to IW. However, in the following sessions, the correlations were high (≥0.8) and significantly different from those before IW (*p* < 0.01 Tukey HSD, η^2^ = 0.38). No significant differences were found in children with CP and TD children after IW (**Figure 5C**).

### Spinal locomotor output in children with severe CP that do not develop IW

We also evaluated the spinal locomotor output in children with severe CP who did not develop IW (group 2). Irrespective of their age (2.8–12 years), they showed characteristics of MN activation strikingly similar to those of much younger children at T0. Thus, they had quasi-synchronous activity of proximal and distal extensors (**Figure 3C**), two optimal clusters of basic activation patterns (**Figure 4C**), and loci of MN activity widely spread across lumbar and sacral segments (**Figure 5D**). Their *CoA* was very poorly correlated (-0.43 ± 0.33) with that of TD children (*p* < 0.01 Tukey HSD, η^2^ = 0.92, **Figure 5D**, see also **Supplementary Figure S4B** for the individual data).

### Neuromechanical considerations

**Figure 6:**
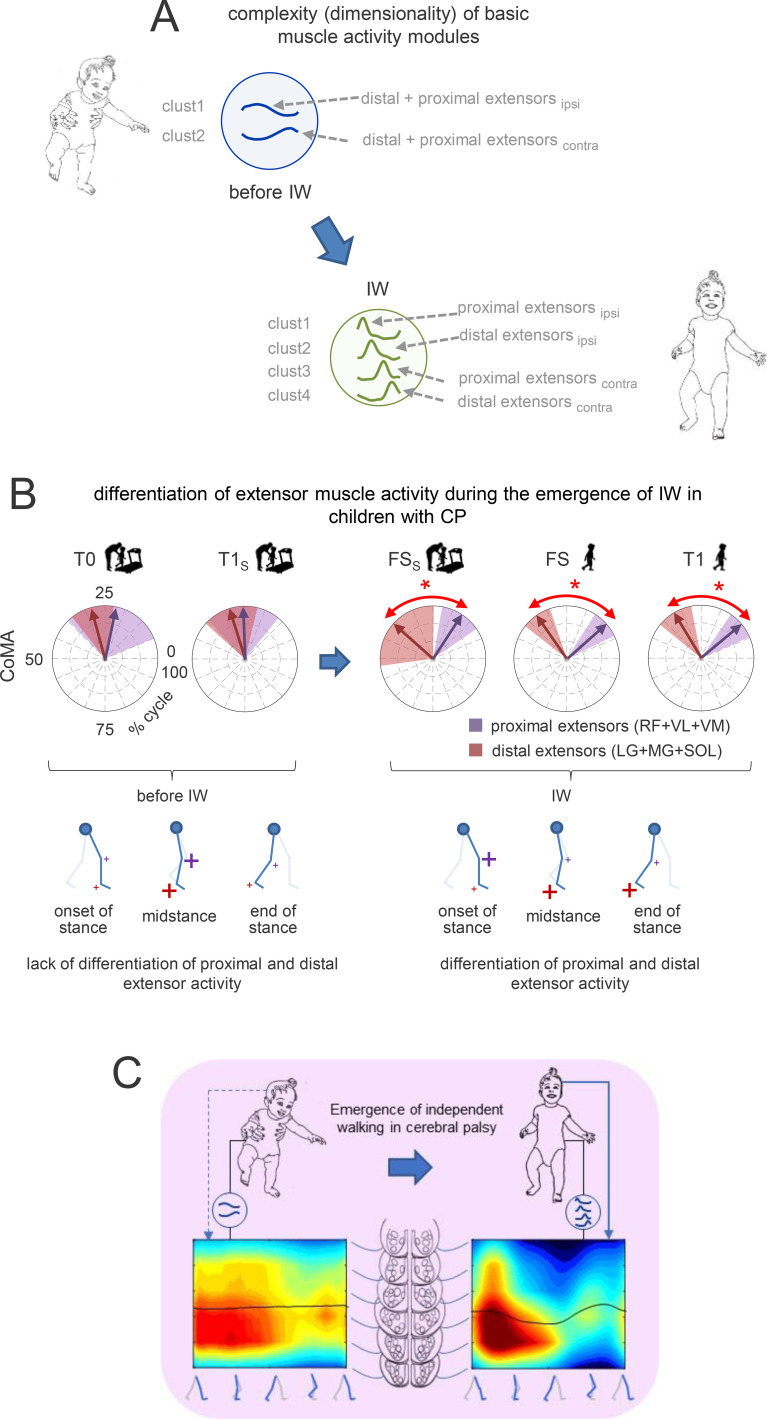
Maturation of the spinal locomotor output in children with CP and neuromechanical correlates. **(A)** Schematic illustration of increasing complexity (dimensionality) of muscle activation patterns during early development. **(B)** Polar plots of the centre of muscle activity (CoMA) of the major groups of extensor muscles. RF, rectus femoris, VL, vastus lateralis, VM, vastus medialis, MG, gastrocnemius medialis, LG, gastrocnemius lateralis, SOL, soleus. Even though some muscles are biarticular and act on different joints, we identified them as proximal (RF+VL+VM) and distal (LG+MG+SOL) extensors. Polar direction denotes the relative time of the CoMA over the gait cycle (time progresses counter-clockwise) and the width of the sector denotes angular SD. Asterisks denote significant differences (circular Watson-Williams test, p<0.05). Note differentiation of extensor muscle activity at the onset of IW. **(C)** Schematic representation of increasing complexity of the spinal locomotor output under the influence of corticospinal inputs during emergence of IW in children with CP.

The augmentation of the neuromuscular modules during early development may have functional significance. Before IW, two basic modules involved coactivation of many leg muscles, while after IW four modules were distributed across specific muscle groups (see synergy bars in **Figure 4A**). **Figure 6A** schematically indicates on which extensor muscles the basic activation patterns loaded. To highlight the functional differentiation of proximal and distal major extensor groups, we analysed changes in their *CoMA*. At T0 and T1_S_, no significant differences in *CoMA* were found, but at FS_S_, FS, and T1, they were clearly differentiated (p<0.001, Watson-Williams test, **Figure 6B**). This differentiation occurred because the *CoMA* of the proximal extensors shifted toward early stance, while that of distal extensors shifted toward late stance, as required for unsupported bipedal gait (Lacquaniti et al., 2012).

## Discussion

We showed that the emergence of mature neuromuscular patterns and synergies was delayed until children with CP took their first unsupported steps (**Figure 4**). The findings also provide insights into the maturation of the spinal segmental output (**Figure 5**) and the functional neuromechanics of the developing spinal locomotor circuitry during the emergence of IW (**Figure 6**), which is discussed below.

### Methodological considerations and motor patterns dimensionality

Mature neuromuscular patterns appeared only after children with CP took their first independent steps (**Figure 4**). It should be noticed that the number of extracted neuromuscular modules should be interpreted as an approximation of the dimensionality of motor control strategies. Indeed, the estimated number of modules is influenced by methodological choices (Zelik et al., 2014; d’Avella et al., 2022; Goudriaan et al., 2022; Sylos-Labini et al., 2022) and should therefore be considered a relative, rather than absolute, measure. In this context, differences in the number of modules between subjects or conditions are more appropriately interpreted as reflecting differences in motor control complexity, rather than the presence or absence of specific physiological modules. Moreover, the estimation of module number may be affected by variability and noise in EMG signals, particularly in paediatric populations characterized by considerable intra-individual (inter-step) and inter-individual variability (Kim et al., 2016; Sylos-Labini et al., 2022). Accordingly, the identified modules should be interpreted as the main representative patterns of motor control organization, rather than the only discrete or invariant statistical entities. Nevertheless, analysing the recorded major leg flexor and extensor groups (that also constitute a relatively large overall lower limb cross-sectional area, Ward et al., 2009) allows capturing the dominant bilateral motor output and the corresponding number of modules (Dominici et al., 2011; Sylos-Labini et al., 2022). To assess the common dimensionality across subjects and/or specific ages, we adopted the analysis by first computing the main basic patterns of individual strides, and then performing k-means clustering.

### Addressing variability and heterogeneity

A primary challenge in paediatric gait analysis, particularly in CP, is the high level of inter-stride and inter-individual variability. To address this issue and increase the robustness of the decomposition, we first pooled the main basic patterns of all strides and all subjects in the group, and then performed k-means clustering. The assumption of such a design is that the patterns common across subjects in each group are the most relevant ones for examining the role of IW in the maturation of motor modules. However, this approach should be interpreted in relation to the specific aim of the present study, which was not to fully characterize subject-specific neuromuscular synergies, but rather to identify robust and physiologically interpretable patterns of spinal motor output that are consistently expressed within and across groups.

While we acknowledge that pooling may bias analysis toward “average” solutions, itprovides a stable representation of the dominant motor output in inherently variable EMG signals. Toaddress this concern, we performed a complementary analysis based on directly extracting modules from the strides of individuals, which confirmed an increase in muscle pattern dimensionality after the onset of IW (**Supplementary Figure S3A**) and reduced dimensionality in group 2 (**Supplementary Figure S3B**). In the analysis, all strides that were not sufficiently close to the cluster centroids were considered mismatched and removed (classified as ‘nc’ although they may contain some gait-relevant patterns albeit they were inconsistent across all strides/subjects). For instance, while some n.c. data in the T1S session appear qualitatively similar to cluster 3 (**Figure 4A**), these patterns did not meet the required threshold for consistency across all strides andsubjects and may represent a small subset of strides, consistent with our individual subjectanalysis where a few participants exhibited three modules rather than two (**Supplementary Figure S3A**, upper panel). Given the heterogeneity of CP pathology, this issue may require further investigation with larger cohorts and/or a larger number of recorded strides.

We used a VAF threshold of 80% for capturing the dominant structure of neuromuscular activation of individual strides, where this level has been shown to capture the main structure of motor control while avoiding overfitting to noise and preserving interpretability of the underlying motor control strategies. Increasing the VAF threshold (e.g., above 90%) typically results in the inclusion of additional components that may reflect noise or minor variability rather than functionally meaningful structure (Sylos-Labini et al., 2020, 2022). For cluster analysis of the individual stride data, the use of a silhouette threshold of S>0.2 reflects the inherent physiological variability and unknown noise levels typical of infant EMG signals, particularly in children with CP. While this threshold indicates modest cluster separation, a more stringent criterion would risk filtering out gait-relevant data. The validity of this choice is supported by the fact that the same clustering parameters consistently captured an increase in dimensionality across both group-level (**Figure 4**) and subject-specific analyses (**Supplementary Figure S3**). The rationale of our approach is based on the presence of the unknown level of noise and considerable intra-individual (inter-step) and inter-individual variability in EMG activity that make it challenging to identify neuromuscular modules in individual infants (Kim et al., 2016; Sylos-Labini et al., 2022). For instance, relying solely on a fixed threshold for the variance accounted for by the selected modules (Steele et al., 2015; Goudriaan et al., 2022) may not be reliable when variable amounts of noise impact the overall output, and correlating such synergy structure between the different gait patterns in children with CP can be problematic (d’Avella et al., 2022; Goudriaan et al., 2022). To mitigate these challenges related to variability and assuming that the patterns common across subjects in each group are most relevant for investigating common age-related maturation processes, we used a criterion evaluating consistent common muscle modules to assess spinal locomotor drive maturation (Kim et al., 2016; Sylos-Labini et al., 2020, 2022) and demonstrated a clear increase in neuromuscular modules after the onset of IW (**Figure 4**).

Other, well established features of motor behaviour in infants with CP, such as the stereotypy oftheir spontaneous movements contrasting with the variability and flexibility of TD infants (Hadders-Algra, 2018; Kwong et al., 2018), are also compatible with the reduced complexity of motor control in CP (Steele et al., 2015; Goudriaan et al., 2022). Finally, we acknowledge that the sample size of the TD and severe CP groups is relatively small, particularly given the complexity of the analytical approach. Nevertheless, the observed patterns were consistent across participants within each group (**Supplementary Figures S2**, **S3**), although these findings should be interpreted with caution and further validated in future studies with larger cohorts.

### Development of basic modules and corticospinal influence

While children with CP and TD children showed comparable complexity when they began walking independently (FS in **Figure 4A**), children with CP experienced a significant delay in maturation, taking their first unsupported steps much later. In CP, two consistent activation patterns appeared in different age groups (**Figure 4B**), suggesting that low-dimensional muscle control persisted until IW irrespective of age. Consequently, the ongoing reorganization of the locomotor output in TD infants during the first year - marked by a steady increase in basic modules (from 2 in newborns to 3 in 6–8 month-olds) (Sylos-Labini et al., 2020, 2022) - is lacking in CP (**Figures 4A, B**), where a shift from two to four basic patterns occurred only after IW onset. Moreover, children with severe CP who were unable to walk independently had characteristics of motoneuron activation strikingly similar to those of much younger children prior to IW.

Overall, these results indicate that IW represents a critical event around which the maturation of motoneuron activation is organized. The emergence of two new patterns during IW (**Figure 4**) may be linked to corticospinal input. First, it is worth noting that IW coincides with emergence of two new patterns also in TD children Dominici et al. (2011). Leonard et al. (1991) hypothesized that “corticospinal pathways may fractionate the infant stepping synergy”, fractionation implying the emergence of new patterns and synergies Zandvoort et al. (2022). This hypothesis is supported by Zandvoort et al. (2022) who found beta-band (13–30 Hz) cortico-muscular coherence for the 2 new synergies in toddlers in cortical regions involving sensorimotor, supplementary motor, and premotor areas (see also Koster et al., 2025). In contrast, cortical sources were absent for the 2 innate synergies. Beta-band cortical oscillations are known to contribute to the control of voluntary movements, locomotion, and posture. Since corticospinal myelination occurs around one year of age in TD children (Eyre et al., 2007; ten Donkelaar et al., 2023), these new patterns likely reflect its maturation. In CP, corticospinal dysfunction correlates with gait impairment (Meyns et al., 2016; Bleyenheuft et al., 2020; Azizi et al., 2021), though intense gait training may induce plastic changes (Willerslev-Olsen et al., 2015). Children with CP who achieve IW, albeit later, likely retain some corticospinal function, allowing the emergence of the new basic patterns (**Figure 6C**). However, the perinatal lesion responsible for CP presumably delays the functional connections between the corticospinal tract and spinal locomotor generators (Yang et al., 2013), resulting in excessive synaptic drive from sensory afferents and hyperreflexia through overstimulation of motoneurons. On the other hand, severe CP cases without IW lack these new neuromuscular modules (**Figure 4C**), presumably due to insufficient corticospinal locomotor connections that do not lead to maturation of the spinal locomotor generators.

While our findings demonstrate a clear association between the onset of locomotor experience (IW) and the maturation of neuromuscular modules, the exact nature of this interaction - whether it is unidirectional or bi-directional - cannot be fully disentangled within the current study design. The observation that older children with severe CP who never walked independently lack these modules may suggest that motor experience is a crucial factor, even though this lack of maturation in group 2 likely reflects both reduced locomotor experience and greater underlying neurological impairment. However, it is plausible that children who achieve IW possess a greater underlying neural capacity that both enables walking and supports the emergence of increased modular complexity. Nevertheless, a reciprocal (bidirectional) interaction between locomotor experience and neural maturation is expected given critical developmental windows for maturation of the supraspinal and spinal circuitries and the necessity for early locomotor rehabilitation (Friel et al., 2014; Hadders-Algra, 2014; Reid et al., 2015; Hurd et al., 2017; Williams et al., 2017). Notice that the onset of IW plays a key role also in important events of typical development, such as the appearance of the pendulum mechanism of walking (Ivanenko et al., 2004).

### Maturation of spinal segmental output

A similar evolution was displayed by the spinal maps of motoneuron output (**Figure 5**). Before IW, children with CP exhibited quasi-synchronous activity of proximal and distal extensors (**Figure 3C**), two optimal clusters of basic activation patterns (**Figure 4C**), and loci of MN activity widely spread across lumbosacral segments (**Figure 5A**). During this period, their CoA was very poorly correlated with that of TD children. However, after IW onset, these loci became more localized and exhibited distinct oscillations (**Figure 5C**, **6C**).

It is important to emphasize that these results represent estimated patterns of spinal motor neurons activity rather than directly measured neural activity. This spinal mapping approach is an indirect reconstruction of motoneuron activity based on surface EMG signals and established anatomical assumptions (Yakovenko et al., 2002; Ivanenko et al., 2006; Avaltroni et al., 2024). Specifically, the method assumes that EMG activity provides an indirect measure of the net firing of the motoneurons (MNs) of a muscle and utilizes myotomal charts to map muscle activity onto the rostro-caudal localization of MN pools in the human spinal cord. While we recorded a limited subset of muscles, and muscle-segment innervation can involve individual anatomical variability, each segment supplies several muscles and each muscle is innervated by several segments, making this subset sufficient to capture the dominant structure of the spinal maps (Ivanenko et al., 2013; La Scaleia et al., 2014).

This approach has been supported and validated in prior work for its physiological plausibility and reproducibility. Originally developed in cats (Yakovenko et al., 2002) and later adapted for humans (Grasso et al., 2004; Ivanenko et al., 2006; Monaco et al., 2010; Wenger et al., 2016), the technique provides an interpretation of motor pool activation at a segmental level rather than at the individual muscle level. Consequently, it serves as a tool to characterize the spinal locomotor output by considering the relative intensities, spatial extent, and temporal structure of motor pool activity in both typical and pathological development, also considering that direct functional imaging of spinal cord activity using magnetic resonance, for instance, is currently limited to stationary participants lying supine in a scanner (Ivanenko et al., 2013; Cappellini et al., 2016; Martino et al., 2018; Avaltroni et al., 2024).

### Neuromechanical considerations for functional augmentation of basic patterns

The augmentation of basic muscle activation patterns may be functional for IW, as unsupported walking requires specific muscle bursts to control the centre of body mass. Muscle activation interacts with neural and mechanical factors, producing pulsatile bursts at specific gait cycle points, which is a distinguishing feature of adult locomotion (Lacquaniti et al., 2012). During supported walking, infants essentially need to compensate for partial body loading, and forward motion is helped by the treadmill belt (or experimenter). However, unsupported walking requires additional muscle bursts for appropriate control of the centre of body mass, and step transitions. At IW onset, proximal and distal extensors show distinct activation (**Figure 6B**), reflecting their roles in weight acceptance and propulsion (Lacquaniti et al., 2012). Thus, augmenting the number of muscle modules is essential for achieving stable, independent bipedal gait. Additionally, musculoskeletal adaptations may also influence these activation patterns. For instance, muscles in children with CP are often weak and atrophic, resulting in significantly reduced muscle volumes and associated bone changes. These factors, along with specific complications such as spasticity, hamstring or equinus contractures (Barrett and Lichtwark, 2010; Moreau et al., 2010; Willerslev-Olsen et al., 2013; Noble, 2014; Handsfield et al., 2016; Goudriaan et al., 2022), contribute to the observed motor profiles, although the relatively young age of the participants in the present study may have limited the presence of severe fixed contractures. We hasten to state, however, that the emergence of new modules after IW in children with CP, roughly similar to those of TD children, does not imply at all a normalization of spinal motor control in CP. Quite to the contrary, several features of locomotion continue to differ drastically from typical walking (Steele et al., 2015; Cappellini et al., 2016; Goudriaan et al., 2022).

## Conclusions and clinical implications

The present findings highlight the interactions between developing nervous system components and contribute to understanding early neurodevelopmental disorders and spinal motor development. While the emergence of IW is strongly associated with the maturation of the spinal output (**Figure 6**), further research is needed to determine the extent to which independent walking serves as a driver of maturation versus a functional outcome of reaching a specific threshold of neural capacity. In this respect, impairments in the modularity of movement control (Lacquaniti et al., 2013) may represent an interesting avenue of research (**Figure 4**) and be a hallmark of early development problems. Also, if the spinal circuitry is compromised due to brain injuries, descending motor pathways presumably control it in a different fashion. This would in turn trigger supraspinal reorganization to compensate, and these reciprocal spinal-supraspinal compensatory mechanisms could increase the risk of irreversible changes in locomotor circuitry and motor cortical areas. Given the existence of critical early maturation periods, our results further support the need for early rehabilitation accelerating the onset of IW (Friel et al., 2014; Hurd et al., 2017). These considerations are pertinent also in the context of the growing importance of societal inclusion of individuals with CP, given the relevance of unaided walking in many work and societal environments (Dan et al., 2025).

## Data Availability

The raw data supporting the conclusions of this article will be made available by the authors, without undue reservation.

## Supplementary material

Supplementary Figure S1 Silhouette values of basic activation patterns and synergies

Supplementary Figure S2 Percentage of strides of individual participants for each activation pattern and synergy

Supplementary Figure S3 Analysis of muscle synergies at the individual level

Supplementary Figure S4 Individual spinal maps
