# Supplementary material for: The role of independent walking in the maturation of the spinal locomotor output in children with cerebral palsy
Source: Front Physiol. 2026 May 13;17:1828195. doi: 10.3389/fphys.2026.1828195 (PMC13212187; doi:10.3389/fphys.2026.1828195)
Supplement: Supplementary Figure S1 — Silhouette values of basic activation patterns and synergies [file DataSheet1.pdf]

Supplementary material

It contains 4 figures:

- Silhouette values of basic activation patterns and synergies (Fig. S1)
- Percentage of strides of individual participants for each activation pattern and synergy (Fig. S2)
- Analysis of muscle synergies at the individual level (Fig. S3)
- Individual spinal maps (Fig. S4)

SILHOUETTE VALUES OF BASIC ACTIVATION PATTERNS AND SYNERGIES

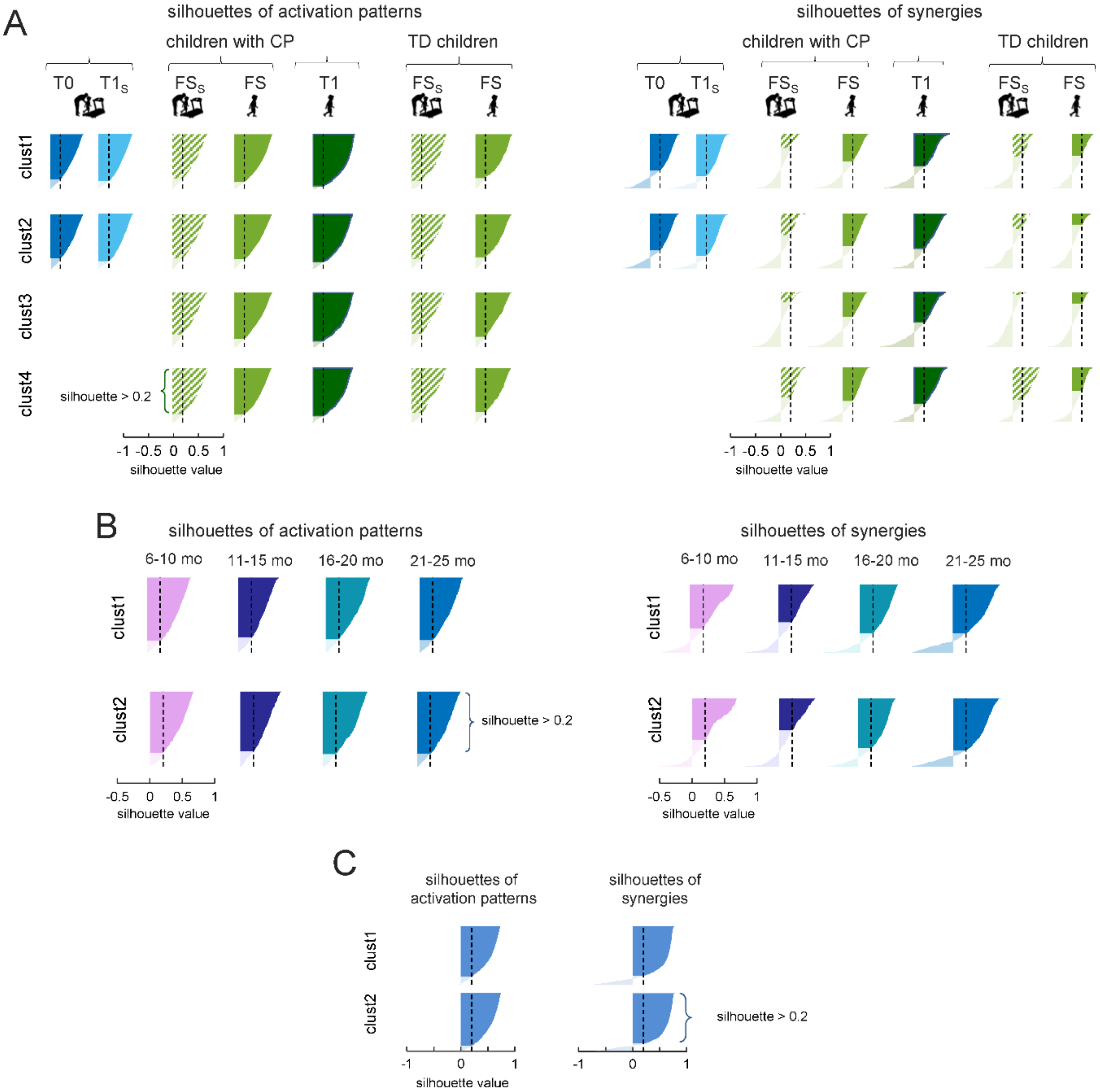

Figure S1. **Silhouette values of basic activation patterns and synergies illustrated in Fig. 2. A**: for all subjects (CP group 1, and TD children). **B:** for children with CP before independent walking. **C:** for children with severe CP that can walk only with external aids (group 2).

Percentage of strides of individual participants for each activation pattern and synergy

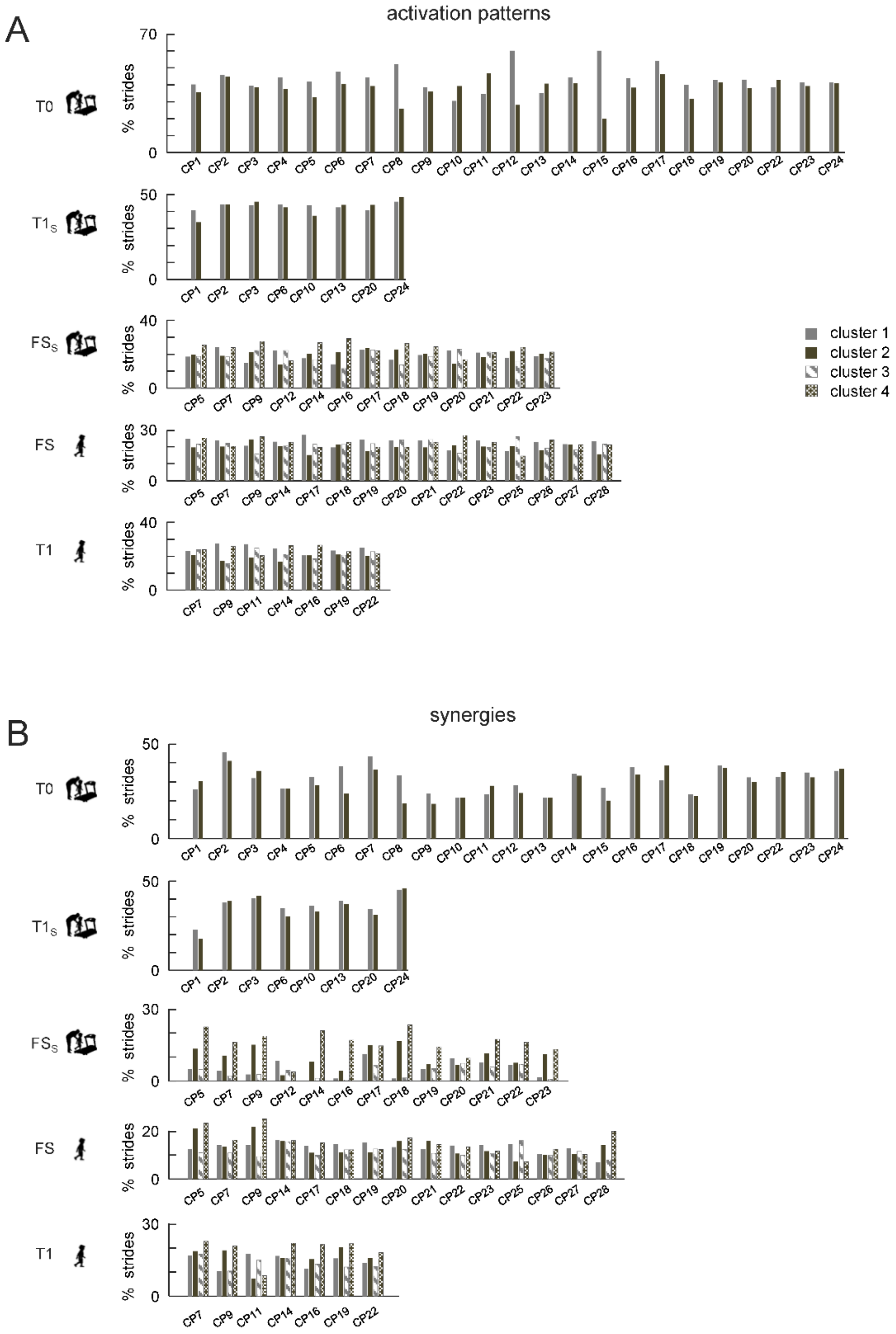

Figure S2. The percentage of strides of individual participants for each activation pattern (A) and each synergy (B) in children with CP. Note relatively uniform distribution of clusters (A) and synergies (B) among participants for each session (T0, $T1_S$, FS_S, FS, T1), indicating that every activation pattern and synergy was present in every child.

ANALYSIS OF MUSCLE SYNERGIES AT THE INDIVIDUAL LEVEL

In addition to reporting the percentage of strides of individual participants for each activation pattern derived by first pooling the basic patterns of all strides and all subjects in the group and then performing k-means clustering, we performed a complementary analysis based on direct extracting the modules from the strides of each individual.

To this end, to account for inter-individual variability, particularly relevant given the heterogeneity of children with CP, instead of pooling all strides across participants, we first extracted muscle synergies separately for each subject, allowing the identification of common modules within individuals and enabling group-level comparisons of synergy-related parameters. We down-sampled EMG signals to 21 points per stride and, for each subject (group 1 and 2), extracted muscle synergies from 18 muscles using non-negative matrix factorization, selecting the minimal number of modules explaining ≥ 80% of the variance. Then, for each subject, we applied k-means clustering (with silhouette analysis, see Methods) to group similar activation patterns across strides, identifying common neuromuscular modules within each subject. Only subjects who performed more than 35 strides were included in the analysis, as EMG signals were time-interpolated to 21 points per stride and an adequate sample size (number of strides > dimensionality of the temporal domain) was required to ensure reliable and stable estimation of muscle synergies.

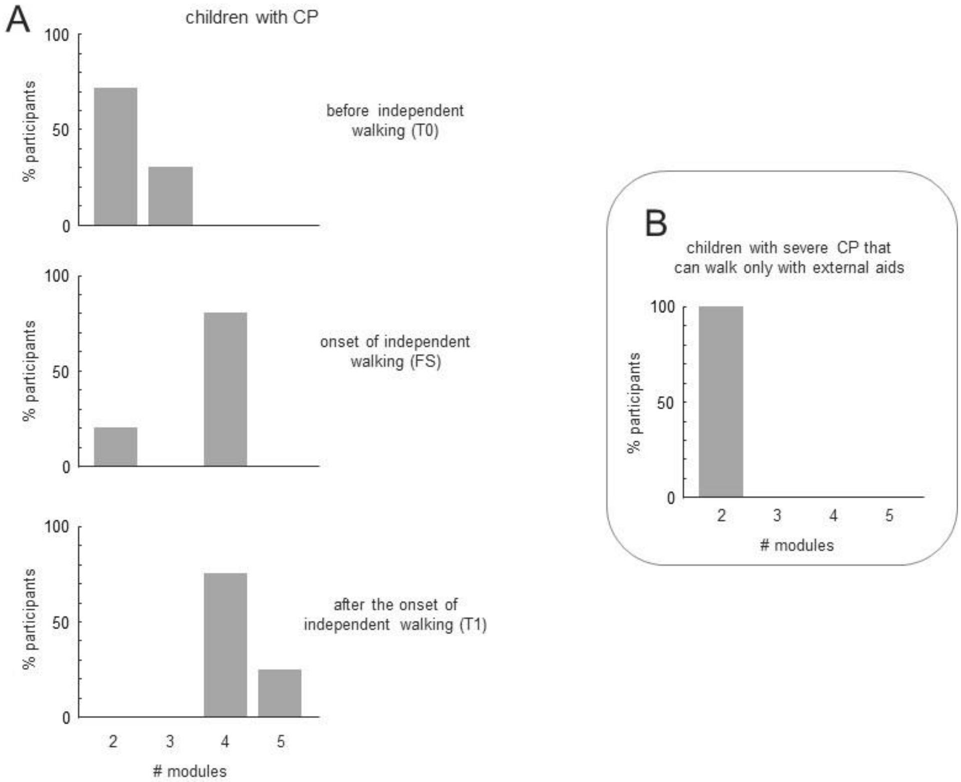

Figure S3. Dimensionality of EMG data using cluster analysis at the individual level. A: group 1 of children with CP.). The percentage of individual participants exhibiting different number of modules before (T0), during (FS) and after IW (T1) in plotted. Note an increase in the number of modules after the IW onset. **B:** group 2 of children with CP.

The results indicate a clear increase in the number of muscle synergy modules across life stages (Figure S3). In group 1 (Figure S3A), before independent walking (IW), the majority of subjects (70%, n=7) exhibited only 2 modules, with few showing 3 modules. During the first steps, a shift toward more complex motor control was evident, as most subjects (79%, n=11) expressed 4 modules, while only 21% (n=3) retained 2 modules. After IW, the pattern stabilized at an even higher complexity, with 75% of subjects (n=3) exhibiting 4 modules and one subject showing 5 modules. In group 2, all subjects (n=4) exhibited only 2 modules (Figure S3B).

In sum, although the methods based only on the %VAF may not be fully suitable for this study given the noise and substantial intra- and inter-individual variability in EMG activity, which complicate the identification of neuromuscular modules in individual infants (d'Avella et al., 2022; Sylos-Labini et al., 2022) (therefore, for the main analyses we used a criterion focused on consistent common muscle modules, Figure 4), these additional findings (Figure S3) confirm that the maturation of locomotor control is accompanied by an increase in muscle synergy dimensionality after the onset of IW, reflecting the emergence of more refined and flexible motor strategies. The observed increase in module number across stages aligns with previous studies reporting that independent walking drives the refinement and diversification of neuromuscular modules (Sylos-Labini et al., 2022).

In addition, we slightly modified our method to allow correlation of synergy dimensionality with the clinical variables (GMFM) listed in Table S1, by assessing the %VAF explained by two synergies (the number prevailing at T0; see Figure S3A). We extracted individual modules using spatial decomposition of averaged EMG data (averaging was used because the number of recorded strides varied greatly across participants, with some children contributing only a few strides) for each child with CP at T0, when GMFM was assessed (Table S1). We then analyzed the correlation between the %VAF of the two-module decomposition and the GMFM at admission. Lower %VAF would indicate that two modules are less sufficient to account for EMG dimensionality. However, no significant correlation was found ($r=-0.04$, $p=0.87$), suggesting that although reduced complexity is often associated with more severe impairment in CP (e.g., Steele et al. (2015); Goudriaan et al. (2022), with exceptions such as Cappellini et al. (2016)), at T0 most children already exhibited reduced dimensionality. Moreover, %VAF (accounting for 2 modules) did not correlate with age at admission ($r=-0.04$, $p=0.84$).

INDIVIDUAL SPINAL MAPS

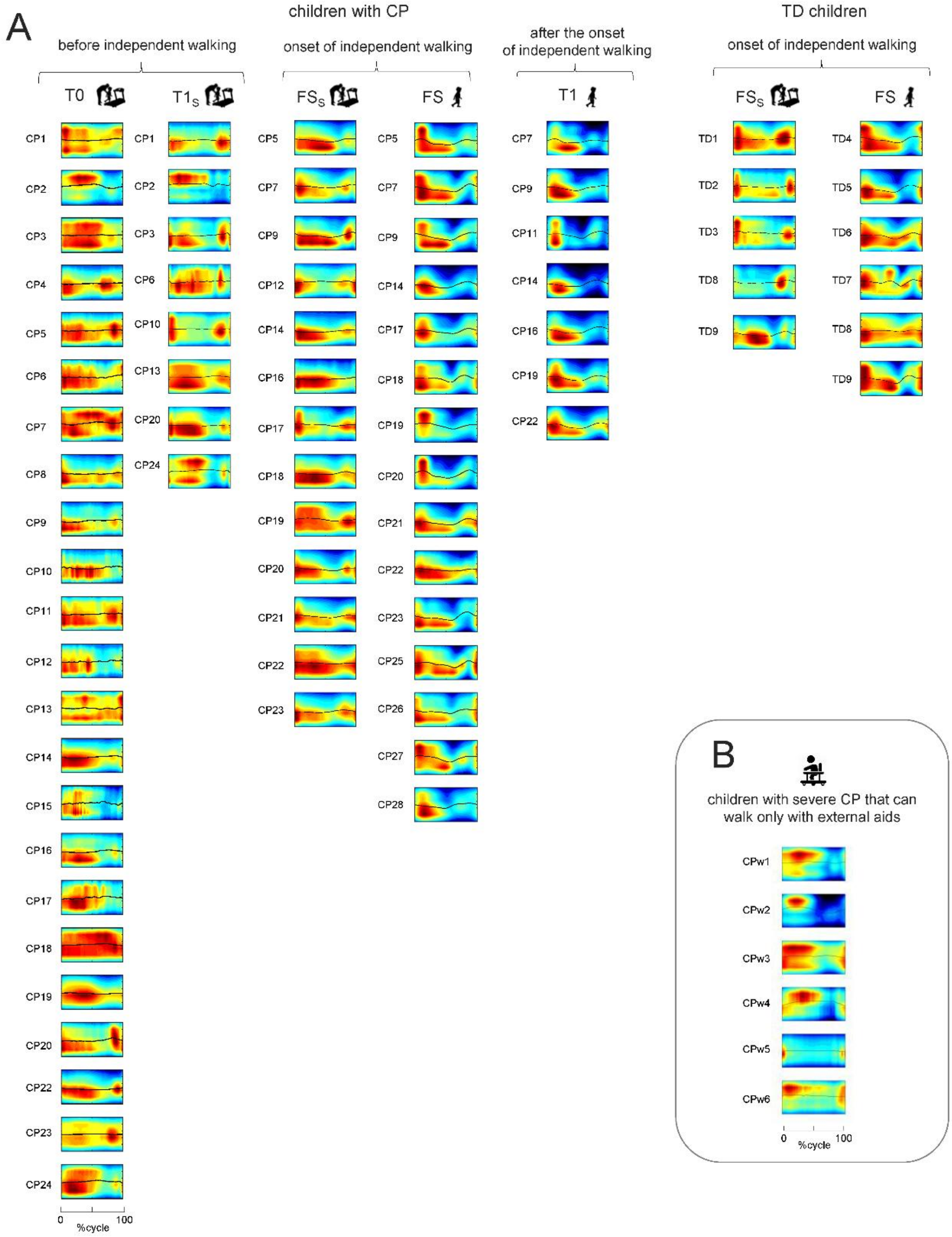

Figure S4. Spatiotemporal maps of motoneuron activity of the lumbosacral enlargement for each child (A: group 1 of children with CP and TD children. B: group 2 of children with CP) and in different experimental sessions. Spatiotemporal unilateral maps of MN activity were reconstructed using EMG envelopes and location of MN-pools in the human spinal cord (Kendall et al., 2005; Avaltroni et al., 2024). Locomotor output for each child was averaged across strides and plotted in a color scale as a function of gait cycle and spinal segment level (L2-S2). To better depict the relative intensities of lumbar and sacral motor pool activation, a color scale (in μV, not shown) was maximized for each child. Black curves indicate the CoA. Similar format as in Fig. 5A.

References

Avaltroni, P., Cappellini, G., Sylos-Labini, F., Ivanenko, Y., and Lacquaniti, F. (2024). Spinal maps of motoneuron activity during human locomotion: neuromechanical considerations. *Front Physiol* 15, 1389436. doi: 10.3389/fphys.2024.1389436

Cappellini, G., Ivanenko, Y. P., Martino, G., MacLellan, M. J., Sacco, A., Morelli, D., et al. (2016). Immature Spinal Locomotor Output in Children with Cerebral Palsy. *Front Physiol* 7, 478. doi: 10.3389/fphys.2016.00478

d'Avella, A., Ivanenko, Y., and Lacquaniti, F. (2022). Muscle synergies in cerebral palsy and variability: challenges and opportunities. *Developmental Medicine & Child Neurology* 64, 404–405. doi: 10.1111/dmcn.15106

Goudriaan, M., Papageorgiou, E., Shuman, B. R., Steele, K. M., Dominici, N., Van Campenhout, A., et al. (2022). Muscle synergy structure and gait patterns in children with spastic cerebral palsy. *Dev Med Child Neurol* 64, 462–468. doi: 10.1111/dmcn.15068

Kendall, F., McCreary, E., Provance, P., Rodgers, M., and Romani, W. (2005). *Muscles. Testing and Function with Posture and Pain*. Baltimore: Lippincott Williams and Wilkins.

Steele, K. M., Rozumalski, A., and Schwartz, M. H. (2015). Muscle synergies and complexity of neuromuscular control during gait in cerebral palsy. *Dev Med Child Neurol* 57, 1176–1182. doi: 10.1111/dmcn.12826

Sylos-Labini, F., La Scaleia, V., Cappellini, G., Dewolf, A., Fabiano, A., Solopova, I. A., et al. (2022). Complexity of modular neuromuscular control increases and variability decreases during human locomotor development. *Commun Biol* 5, 1256. doi: 10.1038/s42003-022-04225-8
